# Infectious Colitis Associated With Ipilimumab Therapy

**DOI:** 10.14740/gr594e

**Published:** 2014-03-14

**Authors:** Jessica L. McCutcheon, Colt M. McClain, Igor Puzanov, Terrence A. Smith

**Affiliations:** a Vanderbilt University Hospital, Department of Medicine, 1161 21st Ave South, D3100 Medical Center North, Nashville, TN 37232, USA; b Vanderbilt University Hospital, Department of Pathology, Microbiology, and Immunology, 1161 21st Avenue South, CC 3322 Medical Center North, Nashville TN 37232, USA; c Vanderbilt University Hospital, Hematology/Oncology Division, 777 Preston Research building Nashville, TN 32232, USA; d Vanderbilt University Hospital, Division of Gastroenterology, Hepatology, and Nutrition, 1301 Medical Center Drive, 1160 The Vanderbilt Clinic, Nashville, TN 37232, USA

**Keywords:** Ipilimumab, Colitis, Infectious diarrhea

## Abstract

Ipilimumab is a monoclonal antibody against cytotoxic T lymphocyte-associated molecule-4 and is thought to promote anti-tumor activity by enhancing cell mediated immunity. It is one of the few therapies shown to improve overall survival in metastatic melanoma. Given its mechanism of action, the drug is associated with significant immune-related adverse events with the gastrointestinal system being commonly involved. Our patient is a 22-year-old female with stage IVA melanoma on ipilimumab therapy who presented with fever, diarrhea and abdominal pain. She gave a history of recent travel to a wedding where several other guests in attendance had also developed diarrheal illnesses. Her colonoscopy and pathology were consistent with ipilimumab-induced colitis. Her stool culture returned positive for *Salmonella enteritides.* She was treated with prednisone and ciprofloxacin with resolution of her symptoms. In our case, we describe ipilimumab-induced colitis where an infectious pathogen was identified with temporal relationship to symptoms and could be suggestive of a causal relationship.

## Introduction

The incidence of malignant melanoma is currently on the rise accounting for the majority of deaths caused by cancers originating from the skin [1]. While metastatic melanoma is a devastating disease, there are a few therapies that can improve survival. Ipilimumab is a monoclonal antibody against cytotoxic T lymphocyte-associated molecule-4 (CTLA-4), which is a strong negative regulator of T cell activation [2]. Ipilimumab is thought to promote anti-tumor activity by enhancing cell mediated immunity and has been shown to improve overall survival in both previously treated and treatment naive patients [3, 4]. Given the drug’s mechanism of action, it is no surprise that treatment with ipilimumab has resulted in significant immune-related adverse events (IRAEs) [5, 6]. In a clinical trial by Hodi et al [3], the incidence of overall IRAEs was reported to be approximately 60% with diarrhea being the most common. Severe immune-mediated enterocolitis occurred in 7% of patients (defined as diarrhea of ≥ 7 stools above baseline, fever, ileus, or peritoneal signs; Grade 3-5) [3]. There are suggestions that increasing doses of ipilimumab may increase the incidence of IRAEs, with grade 3 or 4 diarrhea reported in 18% of patients receiving 10 mg/kg of ipilimumab [7]. Most gastrointestinal IRAEs are reversible with the use of immunosuppressive therapies, but some may result in severe and life threatening events necessitating long term immunosuppression and permanent discontinuation of therapy. Current clinical guidelines also advise to rule out infectious etiologies in patients with gastrointestinal symptoms. We present a case of immune-related severe colitis associated with infectious diarrhea in a patient receiving ipilimumab therapy for metastatic melanoma.

## Case Report

**Figure 1:**
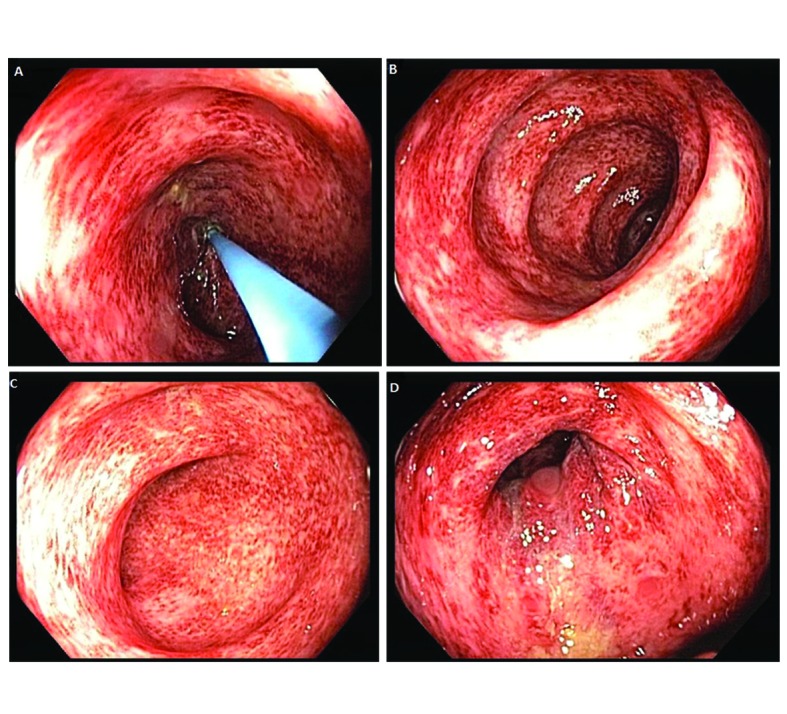
Colonoscopy revealed a grossly normal terminal ileum, mild diffuse right-sided colitis, moderate diffuse mid-colon colitis, and patchy moderate to severe left-sided colitis that was contiguous with the anal canal.

**Figure 2:**
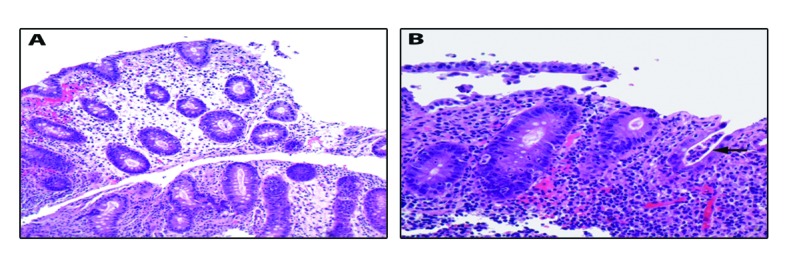
Colonic biopsies were obtained and revealed an intact crypt architecture (A, 200× magnification) with an increased inflammatory cell infiltrate in the lamina propria, neutrophilic cryptitis, crypt abscesses, and surface epithelial injury (B, 400× magnification; arrow highlights crypt abscess).

Our patient is a 22-year-old female with Stage IVA melanoma (BRAF, c-KIT and NRAS negative) of the left arm who is status post resection with positive surgical margins. She underwent local radiation and received immunotherapy with ipilimumab 3 mg/kg every three weeks. She completed three doses of ipilimumab with a rash being her most significant IRAE. The patient presented for admission to our hospital with a one-day history of fever (102.5 F), 10 episodes of large volume watery diarrhea, and mild abdominal discomfort. She gave a history of recent travel to a wedding and noted that two other guests in attendance had also developed diarrheal illnesses. Stool studies were sent for *Clostridium difficile*, bacterial cultures, and ova and parasites. An upright chest film did not reveal any free air under the diaphragm. Ipilimumab was discontinued and high dose prednisone (1 mg/kg once a day) started. Colonoscopy revealed a grossly normal terminal ileum, mild diffuse right-sided colitis with erythema, moderate diffuse mid-colon inflammation without ulceration, and patchy moderate to severe left-colon inflammation without ulceration that was contiguous with the anal canal (Fig. 1A-D). Colonic biopsies were obtained and revealed an intact crypt architecture with an increased inflammatory cell infiltrate in the lamina propria, neutrophilic cryptitis, crypt abscesses, and surface epithelial injury (Fig. 2A, B). Given the clinical setting, these histopathologic features were consistent with ipilimumab-induced colitis. Immunohistochemical staining for Cytomegalovirus was negative. Stool testing was negative for *Clostridium difficile* and ova and parasites. After three days of high dose steroids she continued to have 5 stools per day and her prednisone was increased to 1mg/kg twice a day. On day 4 of her hospitalization, her stool culture was positive for *Salmonella enteritides* and she was started on ciprofloxacin with clinical improvement. She was discharged home on high dose prednisone (1 mg/kg twice a day) with a prolonged taper and a 14-day course of ciprofloxacin. Because her presentation met criteria for severe colitis, ipilimumab was permanently discontinued. At her two-month follow-up, the patient had completed a prolonged steroid taper and reported no further diarrhea. Her most recent Computerized tomography scan revealed multiple new sub-centimeter bilateral pulmonary nodules suspicious for metastatic disease. The remainder of her oncologic treatment course is yet to be determined.

## Discussion

Although ipilimumab is an innovative tool in the treatment of melanoma, the high incidence of IRAEs often limits its use. Kahler and Hauschild report that 84.6% of patients receiving ipilimumab at a dose of 10 mg/kg had drug related side effects with gastrointestinal tract, liver and skin being the most common organs experiencing severe effects [8]. Treatment of severe immune-related enterocolitis requires cessation of ipilimumab and administration of steroids, usually over a prolonged taper of at least one month [7]. If clinically warranted, gastrointestinal perforation should be ruled out. In the setting of refractory colitis, second line therapy involves the use of the anti-TNF monoclonal antibody infliximab. Given the frequency, severity and necessity to discontinue ipilimumab therapy after severe gastrointestinal toxicity, we have a great interest in understanding the pathophysiology of immune-mediated colitis, as well as in developing strategies to manage and prevent this phenomenon.

As of yet, no prophylactic therapies for immune-related enterocolitis have been proven successful. Prophylactic budesonide therapy was evaluated, but not beneficial in reducing the incidence of grade 2 or higher diarrhea [9]. The pathophysiology of immune-related diarrhea is complex with prior studies investigating altered regulation of gastrointestinal mucosal immunity and altered antibody levels to enteric flora in the setting of immune modulating therapy. Although there has been suggestion of many similarities between ipilimumab-induced gastrointestinal toxicity and inflammatory bowel disease, these conditions differ in antibody levels to enteric flora, histologic features and the location of inflammation [10]. It has also been hypothesized that blockade of CTLA-4 can result in depletion of FOXP3+ regulatory T cells, which might explain the mechanism behind immune-mediated enterocolitis [11]. Increased expression of neutrophil activation markers, such as CD177 and CEACAM1, has been associated with risk of gastrointestinal IRAEs in patients receiving ipilimumab. Due to low sensitivity these biomarkers were not felt to be useful for clinical decision making [12].

Gastrointestinal toxicity symptoms often overlap with infectious symptoms and it is still unclear if infectious or enteric flora plays a role in triggering immune-mediated colitis. In our reported case, an infectious pathogen was identified with temporal relationship to symptoms and could be suggestive of a causal relationship. Based on this observation, it may be hypothesized that the use of prophylactic antibiotics could potentially have a preventive effect on the incidence of gastrointestinal IRAEs in patients receiving ipilimumab with hopes of improving the safety profile of ipilimumab. Additional evaluation of the underlying mechanism is necessary to reduce the incidence of drug associated toxicities.
